# Increased resistance of a methicillin-resistant *Staphylococcus aureus* Δ*agr* mutant with modified control in fatty acid metabolism

**DOI:** 10.1186/s13568-020-01000-y

**Published:** 2020-04-07

**Authors:** Hun-Suk Song, Tae-Rim Choi, Yeong-Hoon Han, Ye-Lim Park, Jun Young Park, Soo-Yeon Yang, Shashi Kant Bhatia, Ranjit Gurav, Yun-Gon Kim, Jae-Seok Kim, Hwang-Soo Joo, Yung-Hun Yang

**Affiliations:** 1 Department of Biological Engineering, College of Engineering, Konkuk University, 1 Hwayang-dong, Gwangjin-gu, Seoul, 143-701 Republic of Korea; 2 Institute for Ubiquitous Information Technology and Applications (CBRU), Konkuk University, Seoul, 143-701 Republic of Korea; 3 Department of Chemical Engineering, Soongsil University, Sang-doro, Dongjak-gu, Seoul, 06978 Republic of Korea; 4 Department of Laboratory Medicine, Kangdong Sacred Heart Hospital, Hallym University College of Medicine, Seoul, Republic of Korea; 5 Department of Biotechnology, College of Engineering, Duksung Women’s University, Seoul, 01369 Republic of Korea

**Keywords:** MRSA, *β*-lactam antibiotic, Biofilm, Fatty acid, Surfactant

## Abstract

Methicillin-resistant *Staphylococcus aureus* (MRSA) strains are distinct from general *Staphylococcus* strains with respect to the composition of the membrane, ability to form a thicker biofilm, and, importantly, ability to modify the target of antibiotics to evade their activity. The *agr* gene is an accessory global regulator of gram-positive bacteria that governs virulence or resistant mechanisms and therefore an important target for the control of resistant strains. However, the mechanism by which *agr* impacts resistance to β-lactam antibiotics remains unclear. In the present study, we found the Δ*agr* mutant strain having higher resistance to high concentrations of β-lactam antibiotics such as oxacillin and ampicillin. To determine the influence of variation in the microenvironment of cells between the parental and mutant strains, fatty acid analysis of the supernatant, total lipids, and phospholipid fatty acids were compared. The Δ*agr* mutant strain tended to produce fewer fatty acids and retained lower amounts of C16, C18 fatty acids in the supernatant. Phospholipid analysis showed a dramatic increase in the hydrophobic longer-chain fatty acids in the membrane. To target membrane, we applied several surfactants and found that sorbitan monolaurate (Span20) had a synergistic effect with oxacillin by decreasing biofilm formation and growth. These findings indicate that *agr* deletion allows for MRSA to resist antibiotics via several changes including constant expression of *mecA*, fatty acid metabolism, and biofilm thickening.

## Introduction

*Staphylococcus aureus* is a successful indigenous pathogen in humans, which expresses a wide range of virulence factors, including toxins, immune evasive surface factors, and virulent enzymes (Turner et al. 2019). Moreover, multi-resistant methicillin-resistant *S. aureus* (MRSA) has emerged as a major threat to human health. MRSA strains are generally classified into healthcare-associated MRSA (HA-MRSA) and community-associated MRSA (CA-MRSA), which differ with respect to several characteristics in addition to the source of infection (Mediavilla et al. 2012). HA-MRSA is commonly multi-drug resistant and occasionally causes pneumonia, urinary tract infection, and surgical site infection (Waness 2010). These strains pose a clinical challenge with respect to the difficulty in choice of an appropriate antibiotic for the treatment. In addition, MRSA does not produce the cytotoxin Panton-Valentine leukocidin, and contains type I, II, and III staphylococcal cassette chromosome *mec*. By contrast, CA-MRSA is resistant to β-lactam antibiotics, and causes skin and soft tissue infections along with necrotizing pneumonia (Asghar 2014). However, CA-MRSA strains tend to replicate at much faster rates than HA-MRSA strains, which facilitates their spread (Otter and French 2012). MRSA strains have acquired several mechanisms to inactivate or block the effects of antibiotics. For example, formation of a thick biofilm can act as a physical barrier to antibiotics, and structural differences have also evolved, including variations in membrane composition and modifications of the target of antibiotics (Boudjemaa et al. 2018; Craft et al. 2019; Fishovitz et al. 2014). Therefore, the antibiotic resistance of MRSA poses a significant treatment challenge and gaining greater insight into the mechanisms contributing to this resistance can help to guide treatment and control strategies.

Accessory global regulator (Agr) is a main regulator of gram-positive bacteria (Cheung et al. 2011). In MRSA, the main function of Agr is to regulate biofilm formation and toxin production (Rutherford and Bassler 2012). The *agr* operon acquires signal molecules via a positively regulated mechanism so that RNAII (*agrACDB*) activates the production of phenol soluble modulins (PSMs) and the RNAIII transcripts are also activated to produce δ-hemolysin; moreover, the RNAIII transcript itself controls capsule formation, toxin production, and central metabolism of the strain (Kong et al. 2016).

PSMs are recently discovered short α-helical amphipathic peptides that cause neutrophil lysis after phagocytosis (Chatterjee et al. 2013; Otto 2014). In addition, PSMs have been shown to exert other functions, including acting as regulators in persister cells and biofilm formation (Periasamy et al. 2012; Xu et al. 2017). Following *agr* activation, PSMs are produced to develop the biofilm structure and disperse the biofilm by forming a complex with eDNA, extracellular polysaccharides, and proteins, or by taking advantage of its surfactant characteristics (Schilcher et al. 2016). Regulation of *agr* is also a trigger for changing the antibiotic resistance of MRSA. For example, *agr* expression is somewhat repressed in HA-MRSA strains compared to that in CA-MRSA strains, whereas *mecA* (PBP2a), which has lower affinity to β-lactam, is highly expressed in HA-MRSA and hinders the efficacy of the single use of β-lactam antibiotics (Geisinger and Isberg 2017; Kaito et al. 2011; McCarthy et al. 2015). By contrast, *agr* is highly expressed in CA-MRSA, which results in the production of more toxins rather than becoming resistant to antibiotics as is the case for HA-MRSA (Kaito et al. 2013). A recent study showed that mutation in the *agr* locus led to an increase in biofilm thickness, which might cause higher rates of antibiotic resistance (Periasamy et al. 2012). However, the detailed mechanism by which the *agr* system regulates the resistance mechanism remains unclear.

With the aim of elucidating this mechanism, in the present study, the β-lactam resistance of the *agr* mutant (Δ*agr*) of the MRSA strain *Staphylococcus aureus* USA300-0114 was compared with that of the wild-type strain. Considering that various MRSA strains show stronger resistance with exposure to higher concentrations of antibiotics, we examined the influence of different concentrations of antibiotics that are typically used in microbiology experiments on resistance. To understand the nature of these differences, we further explored the differences in fatty acid distribution and membrane fatty acid composition between the wild-type (WT) and *agr* mutant strains to determine their influence on antibiotic resistance. These findings can provide new insight into the role of *agr* in resistance and guide new strategies for treatment of MRSA infections.

## Materials and methods

### Bacterial strains, media, and culture conditions

For cell preparation, the wild-type strain *Staphylococcus aureus* USA300-0114 (LAC) (CDC 2003) and the Δ*agr* mutant strain (Cheung et al. 2011) was cultured in tryptic soybean broth (TSB) agar and/or liquid broth. For pre-culture, a single colony of the strain from a TSB agar plate was used to inoculate 5 mL of TSB medium. Then, 1% (v/v) of the cell culture suspension was inoculated in a 96-well plate for antibiotic resistance test and cell cultivation was conducted for 24 h in incubator at 37 °C without shaking unless stated otherwise.

### Antimicrobial susceptibility testing

Test for antibiotic sensitivity was conducted by broth microdilution using 96-well plate. To set up MIC assay, twofold serial dilutions of oxacillin, gentamycin, erythromycin, vancomycin, chloramphenicol and teicoplanin were prepared in a microdilution plate. The inoculum was prepared from a sterile swab of colonies from an agar plate to make bacterial suspension which is equivalent to optical density 595 nm of 0.5 McFarland standard. Then, the inoculum was dispensed to microdilution plate and incubated at 37 °C for 24 h without shaking.

### Analysis of cell growth and biofilm formation

For analysis of cell growth and biofilm, 1% (v/v) of the cell culture suspension from the pre-culture was inoculated in a 96-well plate and incubated at 37 °C for 48 h without shaking. Cell growth was measured in terms of cell density, determined according to the optical density using a 96-well microplate reader (TECAN, Switzerland). Biofilm formation was analyzed with crystal violet staining using a previously reported method (Au-O’Toole 2011). Briefly, methanol fixation of biofilm was carried out and 200 μL of 0.2% of crystal violet solution was added to each well and staining was conducted for 5 min for further analysis.

### Analysis of total fatty acids and fatty acids in the supernatant

Gas chromatography–mass spectrometry (GC–MS) was used for the detection and quantification of total fatty acids and supernatant fatty acids, according to a previously described method with slight modification (Bhatia et al. 2017). For total fatty acids analysis, cell cultivation was conducted using 5 mL of TSB with 1% (v/v) inoculum in shaking incubator at 37 °C and 200 rpm. Then, cell was collected at 24 h and 48 h by centrifugation at 3000×*g* for 20 min and washed twice with Milli Q water. The harvested cell was freeze-dried for further methanolysis to analyze total fatty acids. For the analysis of supernatant fatty acids in the medium, spent culture supernatant was incubated in shaking incubator for 2 h at 37 °C and 200 rpm after adding 5 mL of methanol and chloroform to extract fatty acids. The chloroform phase was collected and slowly evaporated under compressed N_2_ at 50 °C in heating block. Fatty acids were re-solubilized with 1 mL of chloroform for further steps. For methanolysis of fatty acids, approximately 10 mg of freeze-dried cells were weighed and placed in Teflon-stoppered glass vials, and then 1 mL chloroform and 1 mL methanol/H_2_SO_4_ (85:15 v/v%) were added to the vials. After incubation at 100 °C for 2 h, the vials were cooled to room temperature, and then incubated on ice for 10 min. After adding 1 mL of ice-cold water, the samples were thoroughly mixed by vortexing for 1 min and then centrifuged at 3000×*g*. The organic phases (bottom of the vials) were extracted by a pipette and transferred to clean borosilicate glass tubes containing Na_2_SO_4_. GC–MS was then performed with a Perkin Elmer Clarus 500 gas chromatograph that was connected to a Clarus 5Q8S mass spectrometer at 70 eV (m/z 50–550; source at 230 °C and quadruple at 150 °C) in EI mode with an Elite 5 MS capillary column (30 m × 0.32 mm × 0.25 μm film thickness; J&W Scientific, USA). Helium was used as the carrier gas at a flow rate of 1.0 mL/min. The inlet temperature was maintained at 300 °C, and the oven was programmed to start at 150 °C for 2 min before increasing to 300 °C at a rate of 4 °C/min, and the temperature was maintained for 20 min. The injection volume was 1 μL, with a split ratio of 50:1.

The structural assignments were based on interpretation of the mass spectrometric fragmentation and confirmed by comparison with the retention times and fragmentation patterns of the authentic compounds along with spectral data obtained from the online libraries of Wiley (http://www.palisade.com) and NIST (http://www.nist.gov). The internal standard was 1 μL of methyl heneicosanoate (10 mg/mL) and Bacterial acid methyl ester (BAME) mix (Merck-Millipore, Burlington, MA, USA) was used to identify each peak of fatty acids and analytical standards for each fatty acid were used for quantification.

### Phospholipid fatty acid analysis

For phospholipid analysis 10 mL of the liquid culture was carried out in shaking incubator using TSB with 1% (v/v) inoculum at 37 °C and 200 rpm. Samples were collected at 8 h and 16 h by centrifugation at 3000×*g* for 20 min, and cell pellet was suspended with 0.15 M citric acid buffer/chloroform/methanol (7:7.5:5 v/v/v) and incubated in the shaking incubator at 37 °C and 200 rpm for 2 h to extract total fatty acids. The chloroform phase was collected, and the chloroform was slowly evaporated under compressed N_2_ to avoid oxidation. The sample was loaded into a sialic column and elution was performed with 5 mL of chloroform, 5 mL of acetone, and 5 mL of methanol serially, and the methanol phase was collected for analysis of phospholipid fatty acids (PLFAs). One milliliter of toluene was added to the sample, which was subjected to mild alkaline trans-methylation with 1 mL of KOH/MeOH at 37 °C for 15 min, followed by cooling to room temperature. A 2-mL aliquot of 4:1 *n*-hexane/chloroform was added, the sample was neutralized with 1 mL of 1 M acetic acid, 2 mL of Milli Q water, and the phases were separated by centrifugation. The upper hexane layer was removed, and this step was repeated with fresh 2-mL aliquots of 4:1 *n*-hexane/chloroform. The combined hexane fractions were concentrated under compressed N_2_ and the fatty acids were re-solubilized with chloroform and analyzed using same method as described above for fatty acid analysis.

### Exogenous surfactants and fatty acids addition test

Surfactants can be used for elimination of contaminating bacteria in medical devices (Hassan and Frank 2003). However, a surfactant itself is not bactericidal but rather modifies the permeability of the cell to be more susceptible to a disinfectant (Walton et al. 2008). Therefore, various surfactants with different properties, ranging from hydrophilic to hydrophobic, were screened to investigate the synergistic effect with antibiotic treatment (Additional file 1: Table S1). Surfactants were solubilized in DMSO for the preparation of 10% (v/v) each stock solution. Pre-culture was conducted inoculating 5 mL of TSB with a single colony from the agar plate, overnight in shaking incubator at 37 °C and 200 rpm. Then, 1% (v/v) of cell was inoculated into the 96-well plate and incubated 48 h at 37 °C without shaking for surfactant and fatty acid effect screening. Surfactants were added to each well at the final concentration of 0.1% (v/v) using 10% (v/v) stock solution for further investigation of their effects. To investigate the fatty acid effects, 100 μg/mL of fatty acids were added to each well as final concentrations using 10 mg/mL stocks in DMSO solution. Antibiotic treatment was carried out using 10 μg/mL and 100 μg/mL of oxacillin for the WT strain and Δ*agr* mutant strain according to their MIC value of oxacillin.

### Reverse transcription for cDNA synthesis and semi-quantitative real time PCR

Pre-culture was conducted by inoculating 5 mL of TSB with a single colony from agar plate, and incubating overnight in shaking incubator at 37 °C and 200 rpm. Cell cultivations were carried out using 5 mL of TSB with 1% (v/v) inoculum in shaking incubator at 37 °C for 12 h and 24 h for RNA extraction. Cells were harvested using centrifuge at 3000×*g* for 20 min. Then, total RNA was prepared using RNeasy Mini Kit (Qiagen, USA) and reverse transcription was performed with Superscript IV Reverse Transcriptase (Invitrogen Co., Carlsbad, CA) to generate the cDNA following the instructions manuals. Primer design was done using Primer express software v3.0.1 from Thermo Fisher Scientific (Waltham, MA, USA), and these primers can generate 150 bp PCR product for the comparison of gene expressions. Before semi-quantitative PCR, cycle number was optimized to set up the saturated gene expression level of *gyrB* (endogenous control) for each template. After optimization, 25 cycle came out to be the optimal cycle number and further comparative analysis of gene expression became possible. Then, Semi-quantitative PCR was conducted using LA taq with GC buffer I (Takara medical co. ltd) using the methods in manual. Primers used for the Semi-quantitative RT-PCR are listed in Additional file 1: Table S2.

## Results

### Increased antibiotic resistance in the Δ*agr* mutant strain

**Fig. 1:**
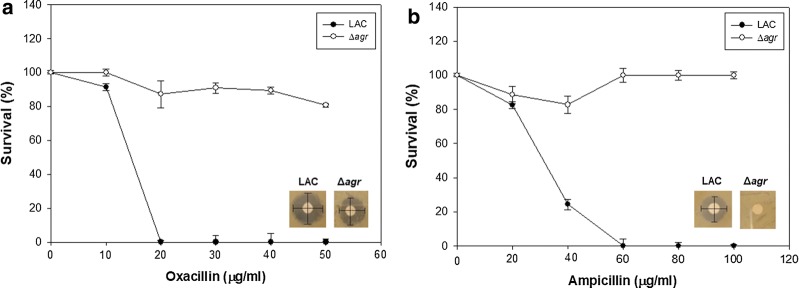
Antibiotic susceptibility test of the LAC and Δ*agr* mutant strain with oxacillin and ampicillin. In the presence of 50 μg/mL of oxacillin and ampicillin the disk diffusion method was carried out. The error bars represent the standard deviation of three replicates

Cell growth was compared between the LAC (WT) strain and Δ*agr* mutant strain under exposure to high concentrations of oxacillin and ampicillin with the paper disc method on agar plate and antimicrobial susceptibility test with 96-well plate. The Δ*agr* mutant strain was able to tolerate 1 μg of ampicillin or oxacillin, whereas the wild-type strain was completely killed with only 0.5 μg of oxacillin or ampicillin on agar plates (Fig. 1a, b). The Δ*agr* mutant strain was able to survive with even higher concentrations of the two antibiotics. Specifically, the Δ*agr* mutant strain could survive 50 μg/mL of oxacillin and 50 μg/mL of ampicillin. Antimicrobial susceptibility test have been conducted to see the difference in antibiotic resistance (Table 1). Especially, the Δ*agr* mutant was able to survive until oxacillin concentration reached 128 μg/mL but LAC (WT) strain did not grow when 32 μg/mL of oxacillin was used. For ampicillin, the Δ*agr* mutant was not inhibited even when the concentration of ampicillin was 1082 μg/mL but LAC was inhibited at 60 μg/mL of ampicillin.

**Table 1:** Antibiotic susceptibility test for LAC WT and LAC Δ*agr* strains

Antibiotics	Disk content	Disk diffusion zone diameter (mm)		Range	Broth microdilution MIC (μg/mL)	
		LAC WT	LAC Δ*agr*		LAC WT	LAC Δ*agr*
Cefoxitin	30 μg	13 (R)	13 (R)	0.5–256	> 32 (R)	> 64 (R)
Teicoplanin	30 μg	16 (S)	15 (S)	1.5–24	3 (S)	6 (S)
Gentamicin	10 μg	25.6 (S)	20 (S)	0.5–8	4 (S)	8 (I)
Erythromycin	15 μg	29 (S)	10 (S)	0.25–128	0.25 (R)	> 64 (R)
Chloramphenicol	30 μg	22 (S)	22 (R)	3–48	12 (I)	12 (I)
Vancomycin	N.I	N.I	N.I	0.5–8	1 (S)	4 (I)
Oxacillin	N.I	N.I	N.I	8–256	> 32 (R)	> 128 (R)
Ampicillin	N.I	N.I	N.I	16–1082	> 64 (R)	> 1028 (R)

### Influence of *agr* on total fatty acids, their distribution, and membrane composition

**Fig. 2:**
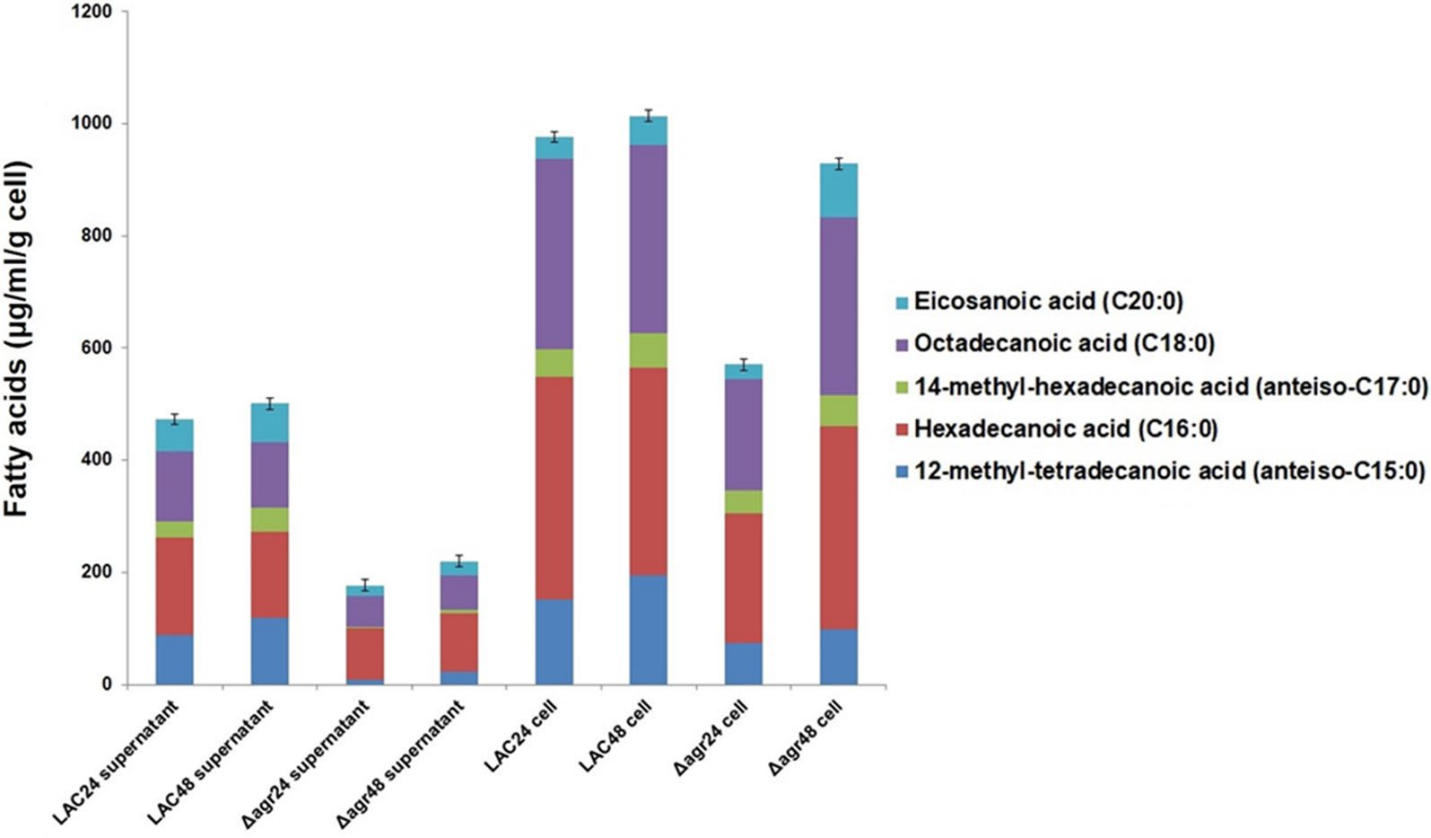
Comparison of total fatty acids and supernatant fatty acid profiles between the LAC and Δ*agr* mutant strain. Supernatant and cell biomass samples were taken separately at 24 h and 48 h. 24 and 48 following strain name indicate the time point of sample collected. The error bars represent the standard deviation of three replicates

**Fig. 3:**
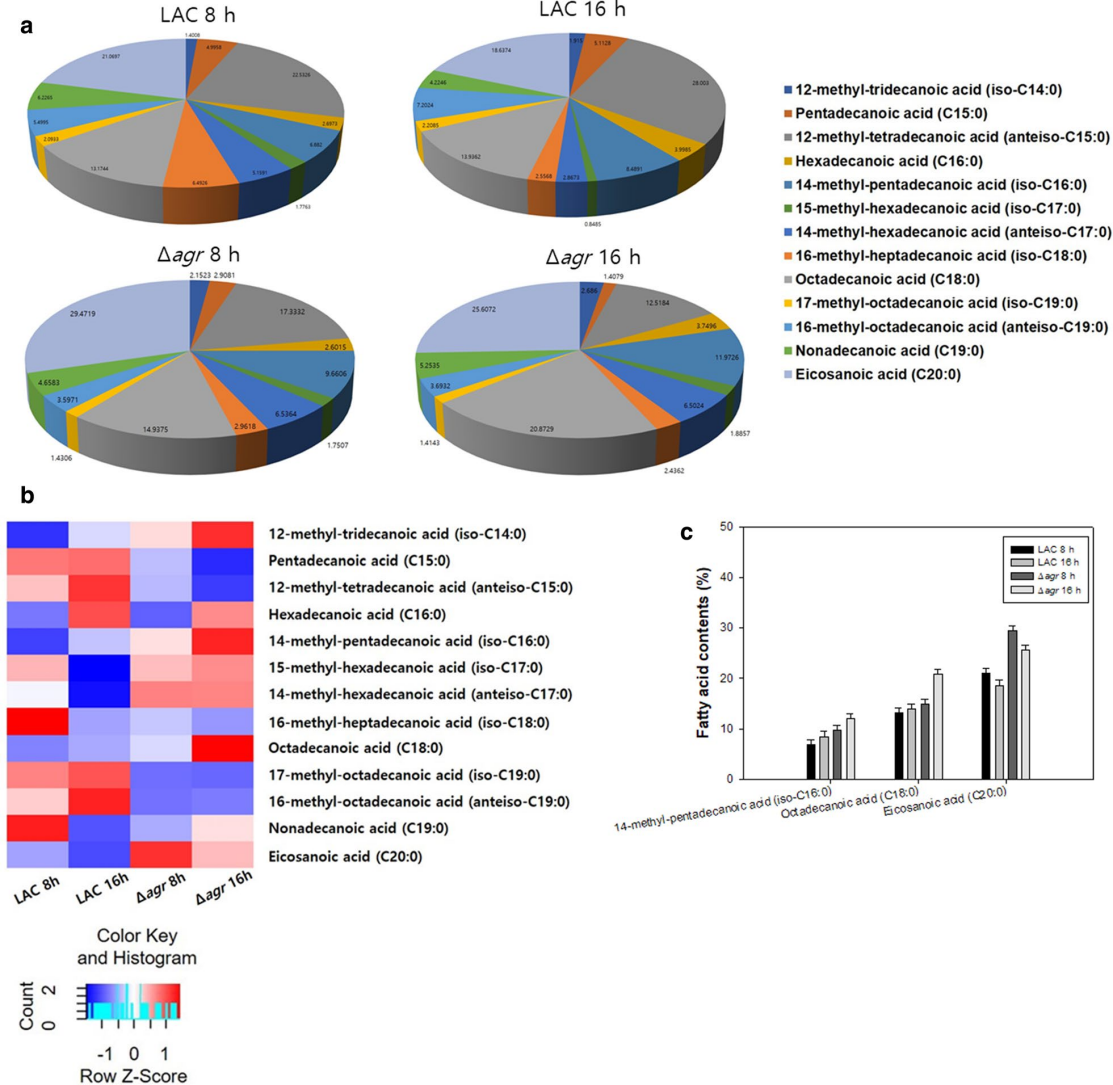
Comparison of phospholipid fatty acids in the LAC and Δ*agr* mutant strains. **a** Compositional analysis of membrane fatty acids at different time points shown as a pie chart. **b** Compositional analysis of membrane fatty acids at different time points shown as a heatmap. **c** Fatty acid contents of 14-methyl-pentadecanoic acid (iso-C16:0) and octadecanoic acid (C18:0) in MRSA. The error bars represent the standard deviation of three replicates

GC–MS results showed that the amount of fatty acids in the Δ*agr* mutant strain during 24 h and 48 h was lower than that of the WT strain. Among major fatty acids in the supernatant, 12-methyl-tetradecanoic acid (anteiso-C15:0), hexadecanoic acid (C16:0), and octadecanoic acid (C18:0) were decreased by the deletion of *agr*. A decrease in total amount of fatty acids was also recorded in the Δ*agr* mutant strain at 24 h, though the difference was not significant between two strains at 48 h (Fig. 2). Phospholipid analysis showed an increase in the portion of fatty acids with longer chains (Fig. 3a, b). Moreover, the portion of 14-methyl-pentadecanoic acid (iso-C16:0), octadecanoic acid (C18:0), and eicosanoic acid (C20:0) in the cytoplasmic membrane were increased in the mutant strain. Long chain saturated fatty acids might have increased the survival rate of the mutant strain to β-lactam antibiotics by decreasing the membrane permeability and increasing the stability of the cytoplasmic membrane (Fig. 3c) (Hashimoto and Hossain 2018; Uzman 2003).

### Influence of the exogenous addition of fatty acids on antibiotic resistance of MRSA

**Fig. 4:**
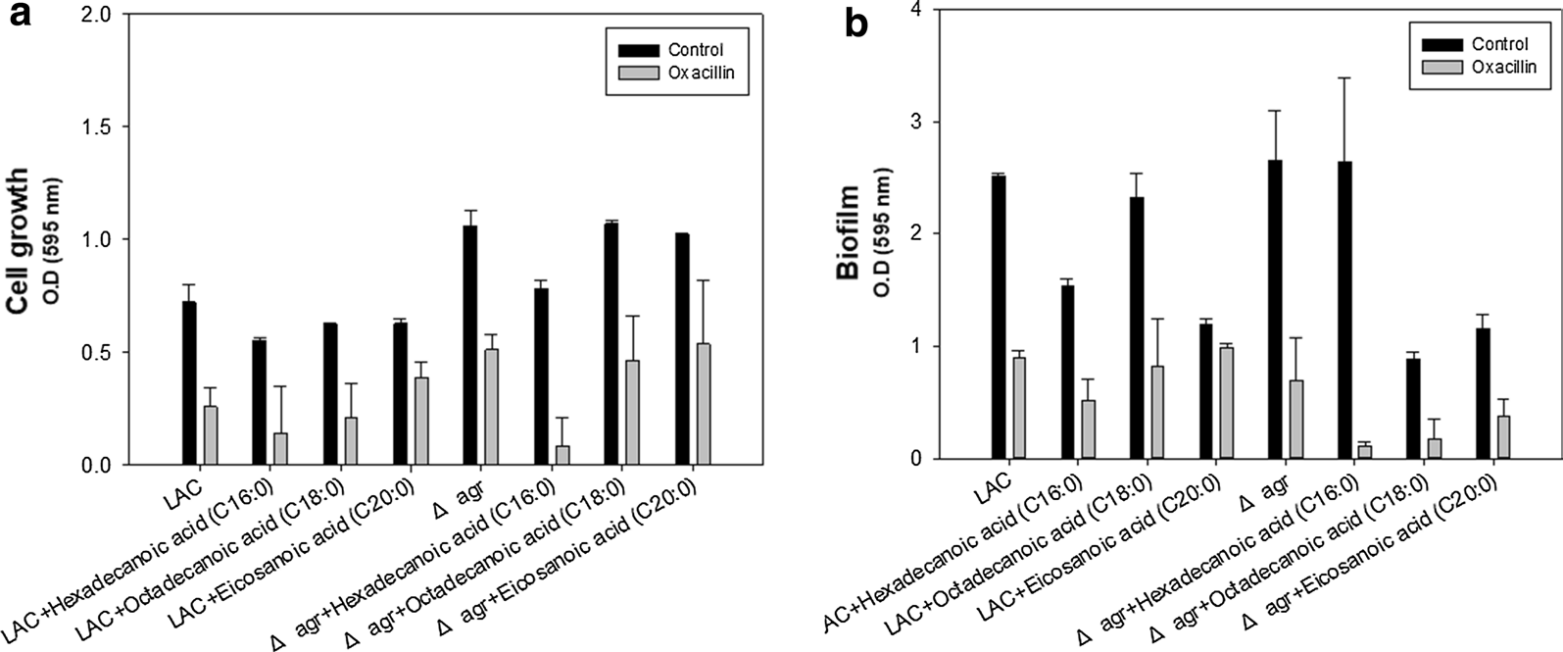
Synergistic effect of oxacillin with artificially added fatty acids. **a** Cell growth of the LAC and Δ*agr* mutant strains with the addition of long-chain fatty acids. **b** Biofilm formation of the LAC and Δ*agr* mutant strains with long-chain fatty acids. The cell was grown for 48 h in the presence of 100 μg/mL of the fatty acid supplements for the comparative analysis of cell growth and biofilm formation. The error bars represent the standard deviation of three replicates

Based on the analysis of fatty acids in the supernatant and phospholipid profiles, we further focused on the influence of C16, C18, and C20 fatty acids which were artificially added to the culture medium to verify the associated changes in antibiotic resistance. Only hexadecanoic acid (C16:0) had a significant anti-bacterial effect with or without oxacillin for both strains (Fig. 4a). All the fatty acids added to the medium without oxacillin inhibited the biofilm formation but the effectiveness was minor with the single use of hexadecanoic acid (C16:0) in the Δ*agr* mutant strain (Fig. 4b). When the Δ*agr* mutant strain was treated with oxacillin in the presence of fatty acids, biofilm formation was greatly reduced but the effect of synergy is not significant in the WT strain. This is because the WT strain has intact *agr* operon and responsiveness of synergism is dependent upon the concentration of oxacillin. One possible explanation is that the WT strain is sensitive to oxacillin and the growth is greatly limited so that the attenuated virulence tends to keep the biofilm formation to survive. Up to now, hexadecenoic acid (C16:0), octadecanoic acid (C18:0) and eicosanoic acid (C20:0) did not show a significant decrease in biofilm formation especially at low concentration (Lee et al. 2017). Hexadecenoic acid (C16:0) even increased the biofilm formation in MRSA strain whereas there was inhibition of biofilm formation with octadecanoic acid (C18:0) (Valliammai et al. 2019). However, hexadecenoic acid (C16:0), octadecanoic acid (C18:0) and eicosanoic acid (C20:0) could reduce biofilm formation in this study. This might be from the different culture condition including medium, bacterial strain and dose-dependent supplementation of fatty acid (Bravo-Santano et al. 2019a; Lade et al. 2019; Yoon et al. 2018). As a conclusion, this phenomenon might explain how the Δ*agr* mutant strain retained a lower level of fatty acid in the supernatant, since maintaining higher levels of fatty acids would interfere with biofilm formation and increase the instability of the cytoplasmic membrane leading to higher antibiotic susceptibility.

### Effect of surfactants on biofilm formation, membrane instability and antibiotic susceptibility

**Fig. 5:**
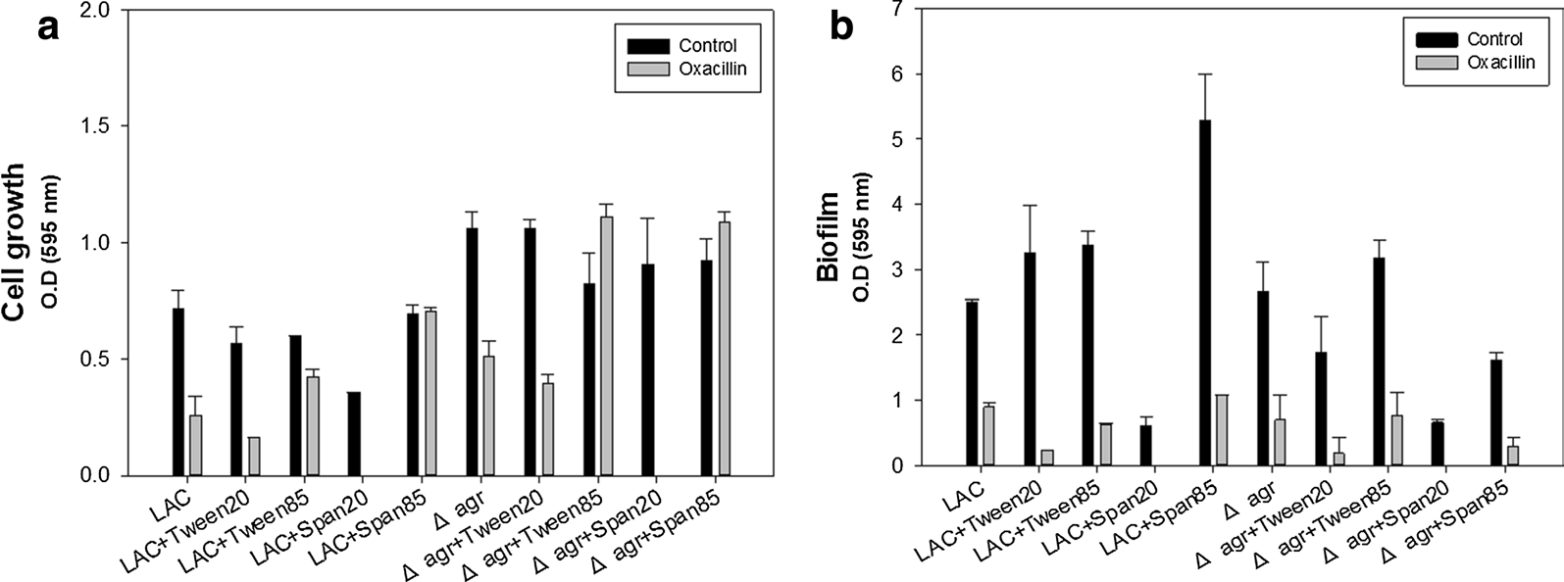
Synergistic effect of surfactants and oxacillin. **a** Cell growth of the LAC and Δ*agr* mutant strains with surfactants. **b** Biofilm formation of the LAC and Δ*agr* mutant strains with surfactants. The cell was grown for 48 h in the presence of 0.1% (v/v) of the surfactant supplements for the comparative analysis of cell growth and biofilm formation. The error bars represent the standard deviation of three replicates

Since the Δ*agr* mutant showed higher resistance to antibiotics along with the formation of a thicker biofilm and higher membrane integrity with long chain saturated fatty acids depending. The effect of surfactant on the susceptibility of MRSA to β-lactam antibiotics was studied. Although the bactericidal effects of the surfactants are not that significant, they can inhibit the cell growth of MRSA. The surfactant span20 has the greatest synergistic effect with oxacillin for both strains (Fig. 5a). Inhibition of biofilm formation was dependent on the culture condition including the strain type, oxacillin and surfactants addition (Fig. 5b), and span20 came out to be the most effective surfactant to eliminate biofilm. This difference might be related to differences in the chemical properties of the surfactants leading to the changes of membrane permeability and instability. Also, membrane microdomain is affected by membrane permeability or integrity so that span20 might have interfered with membrane microdomain assembly reducing the oligomerization of PBP2a thereby increasing antibiotic susceptibility (García-Fernández et al. 2017).

### Changes in *mecA* expression and the fatty acid synthetic operon without antibiotic treatment

**Fig. 6:**
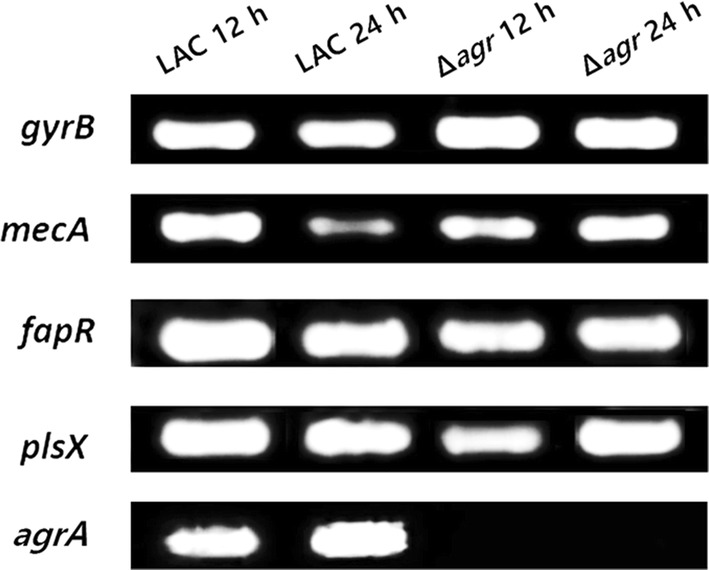
Semi-quantitative RT-PCR of *mecA* and the fatty acid synthetic operon

As mentioned above, HA-MRSA and CA-MRSA show differences in the expression levels of *mecA* and *agr*, which are major factors contributing to increased antibiotic resistance. Moreover, when considering the difference of fatty acid profiles, the expression level of the fatty acid synthetic operon in MRSA strains should be investigated. Therefore, the expression levels of *mecA*, a regulator of fatty acid synthesis *fapR* and the rate limiting step of phospholipid synthesis *plsX* were compared between the LAC and Δ*agr* mutant strains (Albanesi et al. 2013). Semi-quantitative RT-PCR showed that the *mecA* expression level decreased in the WT strain during cultivation but was constantly expressed in the *agr* mutant strain (Fig. 6). In addition, although the *fapR* expression level is lower in mutant strain, the total amount of fatty acids was lower in the mutant strain by total fatty acid analysis. This might be from the fact that malonyl-coA concentration is not enough to interfere with the regulation effect of *fapR* on fatty acid metabolism. Also, it was found that there was lower expression of *plsX* in the Δ*agr* mutant strain. As it is the rate-limiting step of phospholipid synthesis, the total amount of phospholipid decreased in the Δ*agr* mutant strain (Additional file 1: Figure S2).

## Discussion

MRSA is a group of gram-positive bacteria that are genetically distinct from other strains of *S. aureus* and represent a significant public health threat given the limited treatment options for these infections. Agr is a global regulator of MRSA with respect to toxin production, biofilm formation, and cell metabolism (Cheung et al. 2011). Specifically, *agr* is activated to produce toxins increasing virulence during the cell growth and regulated by *mecA* expression when MRSA is exposed to β-lactam antibiotics. There is also a discrepancy between HA-MRSA and CA-MRSA with respect to *mecA* and *agr* expression (Waters et al. 2017), and the Δ*agr* mutant was shown to have a thicker biofilm compared to the WT strain LAC (Periasamy et al. 2012). Thus, we expected a variation of antibiotic resistance depending on the level of *agr* expression and conducted the present study to assess the antibiotic resistance of the *agr* deletion mutant. In addition, considering that various MRSA strains showed stronger resistance with higher concentration of antibiotics, we examined the antibiotic resistance of the mutant and WT strains with exposure to different concentrations of antibiotics that are typically used in microbiology experiments. Specifically, we tested high concentrations of β-lactam antibiotics (> 30 μg/mL), at a level known to kill both methicillin-sensitive *S. aureus* and MRSA. Indeed, the mutant strain could survive at very high levels of the antibiotics, whereas the WT strain was killed at these same levels. Since the Δ*agr* mutant is derived from the LAC (WT) strain, this finding demonstrated that the deletion of *agr* alone could increase the resistance to antibiotics such as oxacillin and ampicillin.

In MRSA*, mec* operon is generally inhibited by *mecI* repressing *mecA* expression to produce PBP2a which confers antibiotic resistance to MRSA. On the other hand, β-lactams increases *mecA* expression by binding to *mecR*1 which in turn cleaves the binding of *mecI* onto the *mec* operon. However, USA300-0114 (LAC) strain contains SCCmec typeIV which is a general type of CA-MRSA, and this type has *mecRI* mutation by insertion sequence. Therefore, the WT strain has constitutively low expression of *mecA* without antibiotic treatment (Rudkin et al. 2014). Along with the fact mentioned above, RT-PCR analysis showed that *mecA* was constantly expressed during cultivation in the Δ*agr* mutant strain increasing resistance to β-lactam antibiotics. In contrast, the WT strain has intact *agr* expression and had limited *mecA* expression at 24 h as is the case for general CA-MRSA strains. However, with deletion of *agr*, the expression level of *mecA* was constant and this suggests that *agr* operon regulate the expression of *mecA*. Nevertheless, the antibiotic resistance mechanism is very complex and involves many more factors in addition to *mecA* expression. Therefore, we next focused on the potential mechanism contributing to the higher survival of the Δ*agr* mutant strain with exposure to much higher concentrations of antibiotics to understand the nature of this antibiotic resistance.

Additionally, we focused on fatty acid metabolism because a previous study showed that the *agr* mutation results in the lack of PSM production and a lower level of fatty acids secretion (Ebner et al. 2017). Additionally, innate immune system in epithelial cell and in abscesses take advantage of long chain free fatty acids such as linoleic acid [C18:2 (n-6,9)], oleic acid [C18:1 (n-9)] and palmitoleic acid [C16:1 (n-7)], which are unsaturated fatty acids and saturated palmitic acid (C16:0) and stearic acid (C18:0) (Bravo-Santano et al. 2019b; Kenny et al. 2009). These major long chain fatty acids are considered bactericidal or bacteriostatic depending on their structural characteristics and they induce stress response of *S.aureus*. Fatty acids are known to have an antibacterial effect owing to their membrane-disrupting activity (Yoon et al. 2018). Therefore, the composition of total fatty acids and extracellular fatty acids were analyzed, and phospholipid fatty acid analysis was conducted with GC–MS. Overall, deletion of *agr* lower the amount of fatty acids in the supernatant and increase the content of long chain fatty acids in the cytoplasmic membrane. Maintaining lower amount of extracellular fatty acids can block the possible anti-bacterial effect from free fatty acids which interfere with electron transfer chain, protein assembly and increase membrane permeability and leakage (García-Fernández et al. 2017; Yoon et al. 2018). On the other hand, accumulation of long chain fatty acids decrease membrane permeability and increase membrane stability (Uzman 2003). These changes might have affected the antibiotic resistance of Δ*agr* mutant strain since physiology of phospholipid affect the antibiotic resistance (Rosado et al. 2015). Moreover, we showed span20 could affect the antibiotic susceptibility of MRSA in a complicated manner by changing membrane stability. With the changes in fatty acid profiles, *mecA* and fatty acid related genes and biofilm collectively led to the increased antibiotic resistance of MRSA. These findings further confirmed that *agr* acts as a regulator for fatty acid metabolism, biofilm and *mecA* expression.

Overall, these results demonstrate the value in understanding the lipid synthesis and secretion of antibiotic-resistant bacteria as a complementary approach to the current focus on resistance genes such as *mecA*, which can provide further guidance toward developing new strategies to overcome antibiotic resistance in MRSA.

## Supplementary information

**Additional file 1.** Additional tables and figures.

## Data Availability

All the datasets on which the conclusions of the manuscript rely are presented in the main paper and additional information.
