# Supplementary material for: Increased resistance of a methicillin-resistant Staphylococcus aureus Δagr mutant with modified control in fatty acid metabolism
Source: AMB Express. 2020 Apr 7;10:64. doi: 10.1186/s13568-020-01000-y (PMC7138893; doi:10.1186/s13568-020-01000-y)
Supplement: Supplementary file 1 — Additional file 1. Additional tables and figures. [file 13568_2020_1000_MOESM1_ESM.docx]

Table S1. Chemical structures and hydrophile-lipophile balance (HLB)

| Surfactant | Chemical structure | HLB |
| --- | --- | --- |
| Tween20 | 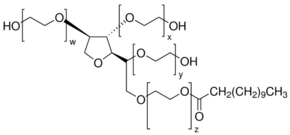 | 16.7 |
| Tween85 | 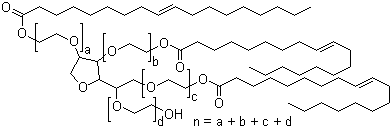 | 11 |
| Span20 | 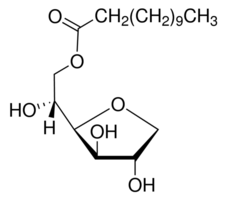 | 8.6 |
| Span85 | 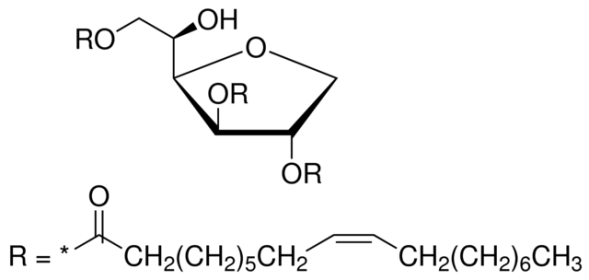 | 1.8 |

Table S2. Primer list used in this study for the semi-quantitative RT-PCR

| Gene | Primer sequence (5’->3’) | Product size (bp) |
| --- | --- | --- |
| *mecA* | F: AGTTAGATTGGGATCATAGCGTCAT | 150 |
|  | R: CGATGCCTATCTCATATGCTGTTC |  |
| *fapR* | F: ATTGCGCGTGGTCATGTG | 150 |
|  | R: TACAACTCGTGCTTCTGCTCTTACC |  |
| *plsX* | F: ATGGCTGAAGCTGTGAAATCTG | 150 |
|  | R:TCCATCAATCGTTGGCAATG |  |
| *gyrB* | F: CGACTTCAGAGAGAGGTTTGCA | 150 |
|  | R: CTGGGATACCACGTCCGTTATC |  |

Fig. S1. Antibiotic susceptibility test of Δ*agr* mutant strain with oxacillin.

Fig. S2. Relative phospholipid fatty acid amount of LAC and Δ*agr* mutant strain.
